# Immune Activation in Pregnant Rats Affects Brain Glucose Consumption, Anxiety-like Behaviour and Recognition Memory in their Male Offspring

**DOI:** 10.1007/s11307-022-01723-3

**Published:** 2022-04-05

**Authors:** Cyprien G. J. Guerrin, Alexandre Shoji, Janine Doorduin, Erik F. J. de Vries

**Affiliations:** Department of Nuclear Medicine and Molecular Imaging, University Medical Center Groningen, University of Groningen, Hanzeplein 1, 9713 GZ Groningen, the Netherlands

**Keywords:** Schizophrenia, Maternal immune activation, Positron emission tomography, Brain glucose consumption, Microglia activation, Anxiety-like behaviour, Recognition memory, Prepulse inhibition

## Abstract

**Purpose:**

Prenatal infection during pregnancy is a risk factor for schizophrenia, as well as for other developmental psychiatric disorders, such as autism and bipolar disorder. Schizophrenia patients were reported to have altered brain metabolism and neuroinflammation. However, the link between prenatal infection, altered brain inflammation and metabolism, and schizophrenia remains unclear. In this project, we aimed to evaluate whether there are changes in brain glucose consumption and microglia activation in the offspring of pregnant rats exposed to maternal immune activation (MIA), and if so, whether these changes occur before or after the initiation of schizophrenia-like behaviour.

**Procedures:**

Pregnant rats were treated with the viral mimic polyinosinic-polycytidylic acid (MIA group) or saline (control group) on gestational day 15. Static PET scans of the male offspring were acquired on postnatal day (PND) 21, 60, and 90, using [11C]-PK11195 and deoxy-2-[18F]fluoro-D-glucose ([18F]-FDG) as tracers to measure TSPO expression in activated microglia and brain glucose consumption, respectively. On PND60 and PND90, anxiety-like behaviour, recognition memory, and sensorimotor gating were measured using the open field test (OFT), novel object recognition test (NOR), and prepulse inhibition test (PPI).

**Results:**

[18F]-FDG PET demonstrated that MIA offspring displayed higher brain glucose consumption in the whole brain after weaning (p = 0.017), and in the frontal cortex during late adolescence (p = 0.001) and adulthood (p = 0.037) than control rats. [11C]-PK11195 PET did not reveal any changes in TSPO expression in MIA offspring. Prenatal infection induced age-related behavioural alterations. Adolescent MIA offspring displayed a more anxious state in the OFT than controls (p = 0.042). Adult MIA offspring showed recognition memory deficits in the NOR (p = 0.003). Our study did not show any PPI deficits.

**Conclusions:**

Our results suggest that prenatal immune activation changed neurodevelopment, resulting in increased brain glucose consumption, but not in microglia activation. The increased brain glucose consumption in the frontal cortex of MIA offspring remained until adulthood and was associated with increased anxiety-like behaviour during adolescence and recognition memory deficits in adulthood.

**Supplementary Information:**

The online version contains supplementary material available at 10.1007/s11307-022-01723-3.

## Introduction

Schizophrenia is a major psychiatric disorder affecting about 1% of the world population [1]. The aetiology of schizophrenia is poorly understood and comprise a combination of genetic vulnerability, and environmental risk factors [2, 3]. Prenatal infection during pregnancy is an environmental risk factor for schizophrenia [4] as well as for other neurodevelopmental disorders, including bipolar disorder and autism [5, 6]. However, the underlying mechanisms linking prenatal infection with the development of schizophrenia remain unclear.

Schizophrenia is associated with brain alterations, such as altered brain glucose metabolism and microglial activation. Positron emission tomography (PET) using the tracer deoxy-2-[18F]fluoro-D-glucose ([18F]-FDG) demonstrated that cerebral glucose uptake is modified in patients with schizophrenia [7, 8]. Post-mortem and PET imaging studies reported glial alterations in the frontotemporal, parietal and hippocampal brain regions [9–11] and increased expression of brain and peripheral inflammatory markers, such as cytokines, in schizophrenia patients [12, 13]. However, none of these studies could provide a direct link between prenatal infection, microglial activation, altered brain glucose metabolism, and schizophrenia. It also remains unclear whether alterations in brain glucose metabolism and microglial activation are the cause or consequence of the development of schizophrenia. To elucidate the relationship between prenatal infection and schizophrenic behaviour, rodent models could be used.

Rodent models of maternal immune activation (MIA) offer a strong face, construct, and predictive validity of, among other neurodevelopmental disorders, schizophrenia [14]. MIA can be achieved by injecting the viral mimic polyinosinic: polycytidylic acid (poly-I:C) in pregnant rats [14]. Imaging studies observed that offspring from rats injected with poly-I:C during pregnancy displayed an altered brain glucose consumption, as measured by [18F]-FDG PET, in the amygdala, hippocampus, and prefrontal cortex [15, 16], and higher translocator protein (TSPO) levels in the prefrontal cortex and hippocampus of MIA offspring using [11C]-PK11195 PET, indicative of the presence of neuroinflammation [17]. The offspring of MIA rats display altered behavioural phenotypes relevant to the positive, cognitive, and negative domains of schizophrenia [18–22]. Notably, studies observed that MIA offspring have impaired prepulse inhibition (PPI), a measure of sensorimotor gating [23]. In addition, MIA offspring show impaired recognition memory in the novel object recognition (NOR) test [24, 25]. Lastly, anxiety-like behaviour in the open field test (OFT) and elevated-plus maze test [19, 21] is observed in MIA offspring. However, whether the behavioural alterations in the MIA offspring are related to changes in brain glucose consumption and glial activation remains unclear.

In this study, we aimed to evaluate whether there are changes in brain glucose consumption and microglial activation in the offspring of pregnant rats exposed to MIA. Furthermore, we also wanted to determine whether the changes in brain glucose consumption and microglial activation occur before or after the initial development of behavioural alterations induced by MIA. To do so, we performed PET imaging at different time points using the PET tracers [18F]-FDG and [11C]-PK11195 for measuring brain glucose consumption and translocator protein (TSPO) levels specific aspects of metabolism and neuroinflammation respectively, in offspring from pregnant rats exposed to poly-I:C. We selected PPI, NOR and OFT tests to measure sensorimotor gating, recognition memory and anxiety-like behaviour, symptoms observed in schizophrenia and other neurodevelopmental disorders such as autism and bipolar disorders. These tests allow measurement of the positive, cognitive, and negative domains of schizophrenia and were performed for validation of the MIA model and to determine possible association with the PET imaging. These tests were performed in adolescence and adulthood.

## Materials and Methods

### Animals

All procedures described in the present study were performed according to European Directive 20,100/63/EU and the law on animal experiments in the Netherlands. Eight 3-month old pregnant female Wistar rats (gestational day 7) were obtained from Harlan, The Netherlands, and were individually housed with ad libitum access to food and water. After arrival, the rats were acclimatized for at least seven days. Housing rooms were humidity-controlled and thermo-regulated (21 ± 2 oC), with a 12:12-h light:dark cycle (lights on at 7 a.m.). Only males were included to prevent oestrous cycle variation, which was previously shown to affect the outcome of [18F]-FDG PET [26].

### Prenatal Immune Activation

On the gestational day (GD) 15, 8 pregnant dams were anaesthetized with 5% isoflurane in oxygen and intravenously injected with either 4 mg/kg poly-I:C in saline (MIA) or saline (control). Prenatal immune challenge in late gestation (GD15) using 4 mg/kg of poly-I:C is a timing and dosage commonly reported in rats [27] that corresponds to the migration of cortical neurons, myelination, neurogenesis and synaptogenesis [28]. Poly-I:C (sodium salt; Sigma-Aldrich, Schnelldorf, Germany) was dissolved in 0.9% NaCl solution to yield a final concentration of 2 mg/ml. All solutions were freshly prepared on the day of administration. Once awake, all animals were returned to their home cages. A guideline checklist for the methodological details regarding the MIA model can be found in the supplemental materials [29]. To reduce the litter effect, two male offspring per litter were used.

### Study Design

**Fig. 1.**
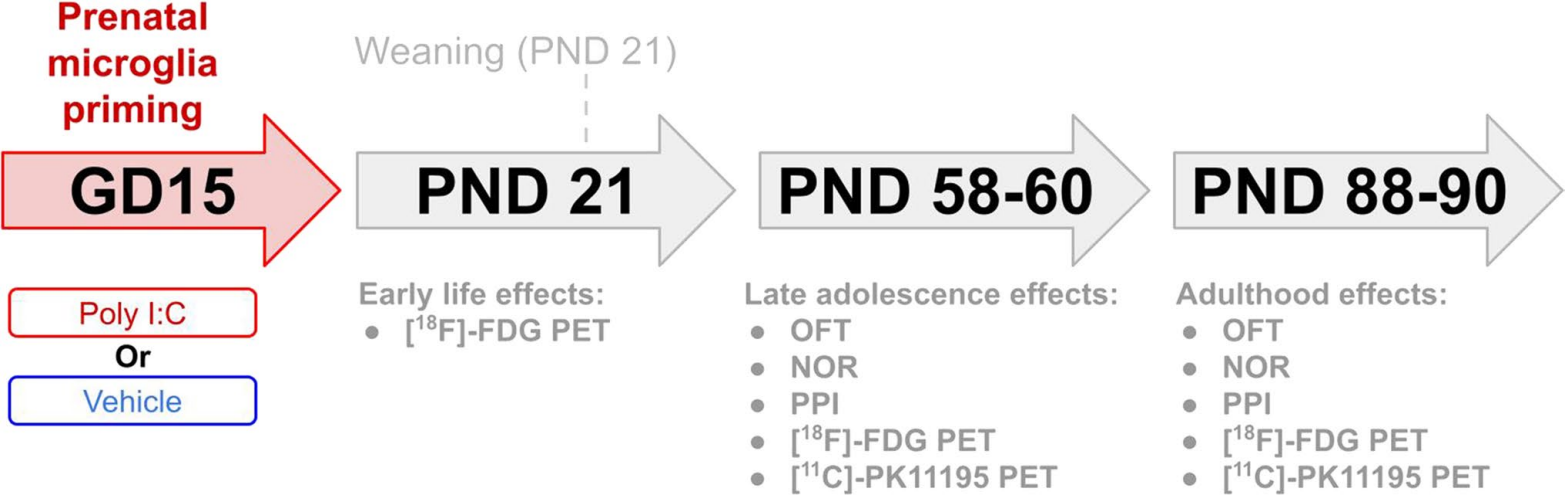
Study design. Pregnant dams were intravenously injected with either saline or poly-I:C on gestational day (GD) 15. Male offspring were weaned on postnatal day (PND) 21. Open field test (OFT) and novel object recognition test (NOR) were performed on PND58 and 88. Prepulse inhibition (PPI) measurements were performed on PND59 and 89. [18F]-FDG PET scans were performed on PND21, 60, and 90. [11C]-PK11195 PET scans were performed on PND60 and 90.

Sixteen male offspring were randomly divided into two groups: (1) offspring from pregnant dams injected with vehicle (control), (2) offspring from pregnant dams injected with poly-I:C (MIA). Male offspring were weaned on PND21 and animals from the same litter were group-housed. Behavioural tests to measure anxiety, memory, and sensorimotor gating were performed on PND58-59 and PND88-89 (Fig. 1). PET scans to measure brain glucose consumption were performed on PND21, 60 and 90 and PET scans to measure microglial activation on PND60 and 90.

### Open Field Test

The open field test (OFT) was performed on PND58 and 88 to measure anxiety-like behaviour and locomotion. Rats were allowed to acclimatize to the experimental room for 2 h before being placed in an ellipsoid arena (126*88 cm) for 5 min. The time spent in the centre (an ellipsoid area at ≥ 20 cm from the wall) was measured to determine the level of anxiety-like behaviour. Behaviour was recorded on video and analysed offline using Ethovision XT 8.5 (Noldus Information Technology, Wageningen, The Netherlands). The arena was cleaned with 70% ethanol solution after each session.

### Novel Object Recognition Test

Short-term recognition memory was assessed with the novel object recognition test (NOR). Rats were allowed to acclimatize to the experimental room for 2 h. The test consisted of three phases: the habituation, familiarization, and test phases. In the habituation phase, the rat was placed in an open arena (50*50*50 cm) and allowed to habituate for 10 min. In the familiarization phase, the rat was permitted to freely explore two identical objects in the same arena, before being returned to its home cage for 2 h. In the test phase, the rat was put back into the arena for 5 min, but one of the objects was replaced by a novel object. Behaviour was video recorded and the time exploring each object was manually analysed. A discrimination index was calculated by dividing the time spent with the novel object by the total time spent investigating both objects. The arena was cleaned with 70% ethanol solution after each session. Rats that stayed immobile for more than 180 s were excluded. On PND58, 2 control and 3 MIA rats were excluded for this reason. On PND88, 3 control and 1 MIA rats were excluded.

### Prepulse Inhibition Test

A prepulse inhibition test was performed to measure alterations in sensorimotor gating. The principle of this test is that a weaker pre-stimulus (prepulse) inhibits the reaction to a subsequent stimulus (pulse). The test was performed in an acoustic startle chamber mounted with a piezoelectric accelerometer to detect whole-body startle response (TSE systems, Germany). First, the rat was placed in the startle box to acclimatize for 5 min with a background noise of 70 dB (white noise). The background noise was present during the entire session. The habituation phase consisted of 3 startle pulses alone to allow the rat to get familiar with the sound of the pulse. After habituation, the test session consisted of 8 × 4 trials in random order: (1) only pre-pulse, 85 dB sound for 20 ms; (2) only startle pulse, 120 dB sound for 40 ms; (3) pre-pulse and startle pulse, 85 dB pre-pulse for 20 ms followed 100 ms later by 120 dB sound for 40 ms; (4) no sound, only 70 dB background noise.

### PET Imaging

Small animal PET (microPET Focus 220, Siemens) was used to measure brain glucose consumption on PND21, 60 and 90 and TSPO levels following microglial activation on PND60 and 90. Rats were either intraperitoneally injected with [18F]-FDG (13.9 ± 5.6 MBq) or briefly anaesthetized with 5% isoflurane in medical air for an intravenous injection of [11C]-PK11195 (9.3 ± 5.6) in the tail vein. There was no difference in injected tracer dose between groups. After tracer injection, rats were placed back in their home cage. About 35 min after tracer injection, rats were anaesthetized with isoflurane (induction 5% and maintenance 2%, in medical air) and positioned into the PET camera for a transmission scan of 10 min followed by an emission scan of 30 min, starting at 45 min after tracer injection. The body temperature of the rat was maintained with heating pads, blood oxygen levels were monitored, and an eye salve was applied to prevent dehydration. After the scan, rats were placed back into their home cage to recover. After the last scan on PND90, the rats were terminated under deep anaesthesia by heart extirpation. [11C]-PK11195 PET was carried out in the morning and [18F]-FDG PET in the afternoon.

PET scans were iteratively reconstructed (OSEM2D, 4 iterations, 16 subsets) into a single frame, resulting in images with a 128 × 128x95 matrix, a pixel width of 0.632 mm, and a slice thickness of 0.762 mm. PET images were automatically co-registered to a functional [18F]-FDG or [11C]-PK11195 template [30], which was spatially aligned with a stereotaxic T2-weighted MRI template in Paxinos space [31]. The co-registered images were resliced into cubic voxels (0.2 mm) and converted into standardized uptake value (SUV) images, assuming a tissue density of 1 g/ml (SUV = [tissue activity concentration (MBq/ml) x body weight (g)]/ [injected dose (MBq)]). Tracer uptake was calculated in volumes-of-interest (VOI) representing specific brain regions. Due to the limited resolution of the small animal PET scanner (1.4 mm) [32], small brain regions were excluded [33].

### Statistical Analysis

Statistical analysis of body weight, behaviour, and PET data was performed using SPSS (IBM SPSS Statistics, Version 22.0). A generalized estimating equation (GEE) analysis, using ‘maternal infection’ and ‘time’ as factors, was performed for the statistical analyses of longitudinal data, as this analysis can account for missing data. Data are presented as mean ± standard deviation (SD).

## Results

### MIA Decreased Body Weight at Weaning

**Fig. 2.**
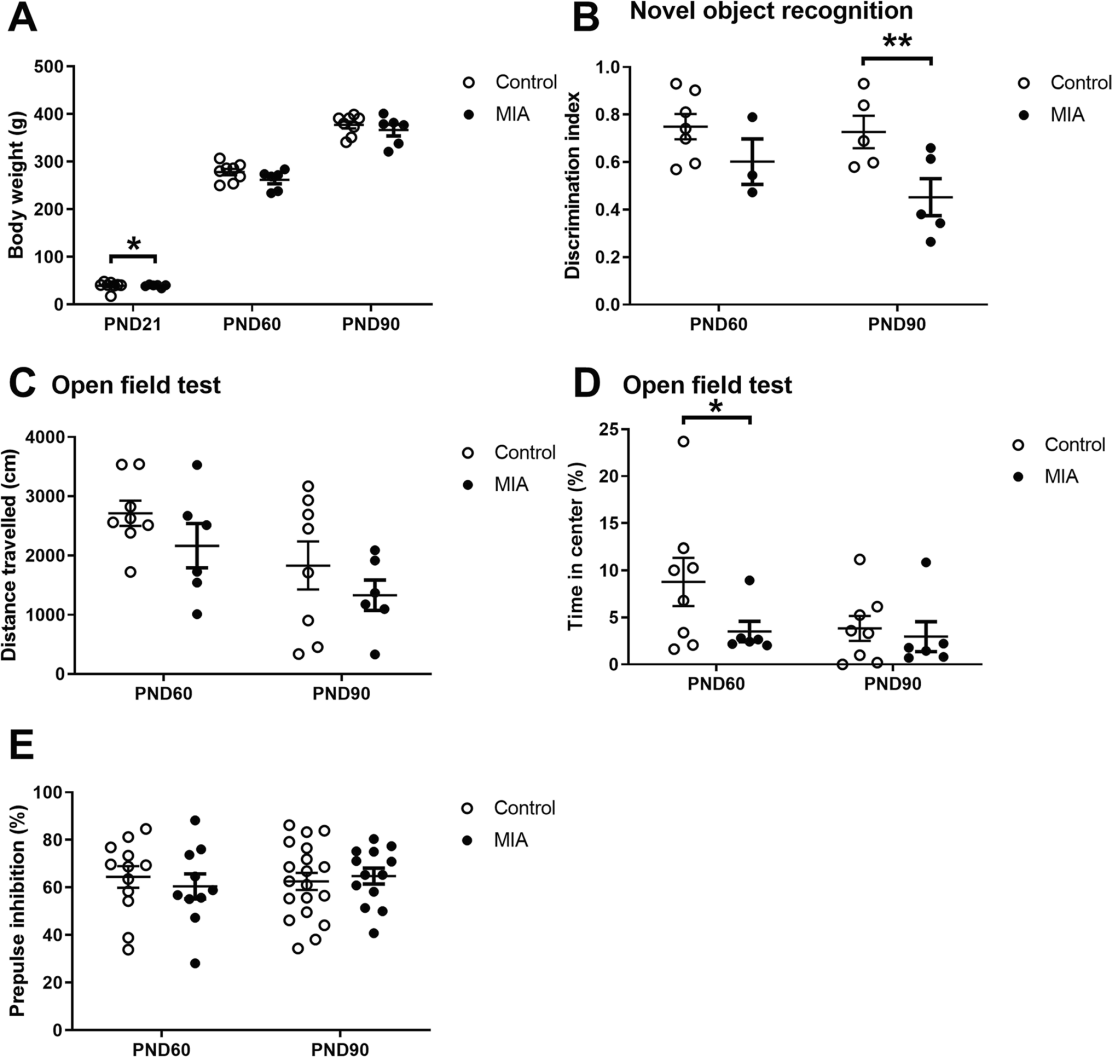
Bodyweight and behavioural changes**. A.** Bodyweight (control: n = 8, MIA: n = 6.) **B.** Recognition memory (PND60: control n = 6, MIA: n = 3, PND90: control: n = 5, MIA: n = 5). **C.** Locomotion (control: n = 8, MIA: n = 6). **D.** Anxiety-like behaviour (control: n = 8, MIA: n = 6). **E.** Prepulse inhibition (PND: control: n = 12, MIA: n = 10, PND90: control: n = 19, MIA: n = 13). PND = postnatal day. Values represent mean ± SD. Statistically significant differences between groups are indicated by asterisks: *P < 0.05, **P < 0.01, ***P < 0.001. Significant differences between time points are not shown.

The bodyweight of both control and MIA rats increased over time (p < 0.0001, Fig. 2A). At PND21, the bodyweight of MIA offspring was significantly lower when compared to controls (-9.1%, p = 0.014), but this difference was no longer present on PND60 (p = 0.104) and PND90 (p = 0.467).

### MIA Caused Recognition Memory Deficits on PND90

The discrimination index in the NOR test was used to assess recognition memory (Fig. 2B). Offspring from mothers exposed to MIA displayed a significantly lower discrimination index than controls on PND 90 (-38%, p = 0.003), but not on PND60 (p = 0.10).

### MIA Caused Anxiety-like Behaviour on PND60 but did not Affect Locomotion

The total distance travelled in the OFT and the percentage of time spent in the centre of the arena were used to assess locomotion (Fig. 2C) and anxiety-like behaviour (Fig. 2D), respectively. The distance travelled decreased over time in both groups (p = 0.004) but did not significantly differ between groups on PND60 (p = 0.16) or PND90 (p = 0.26). MIA rats spent significantly less time spent in the centre of the arena than controls on PND60 (-60%, p = 0.042), but not PND90 (p = 0.61).

### MIA did not Modify PPI

The percentage of PPI was used as an indicator of sensorimotor gating (Fig. 2E). We observed no main effect of time within groups (p = 0.731). There was no difference in PPI between MIA and control offspring on PND60 (p = 0.56) or PND90 (p = 0.65).

### MIA did not Induce Microglial Activation

**Fig. 3.**
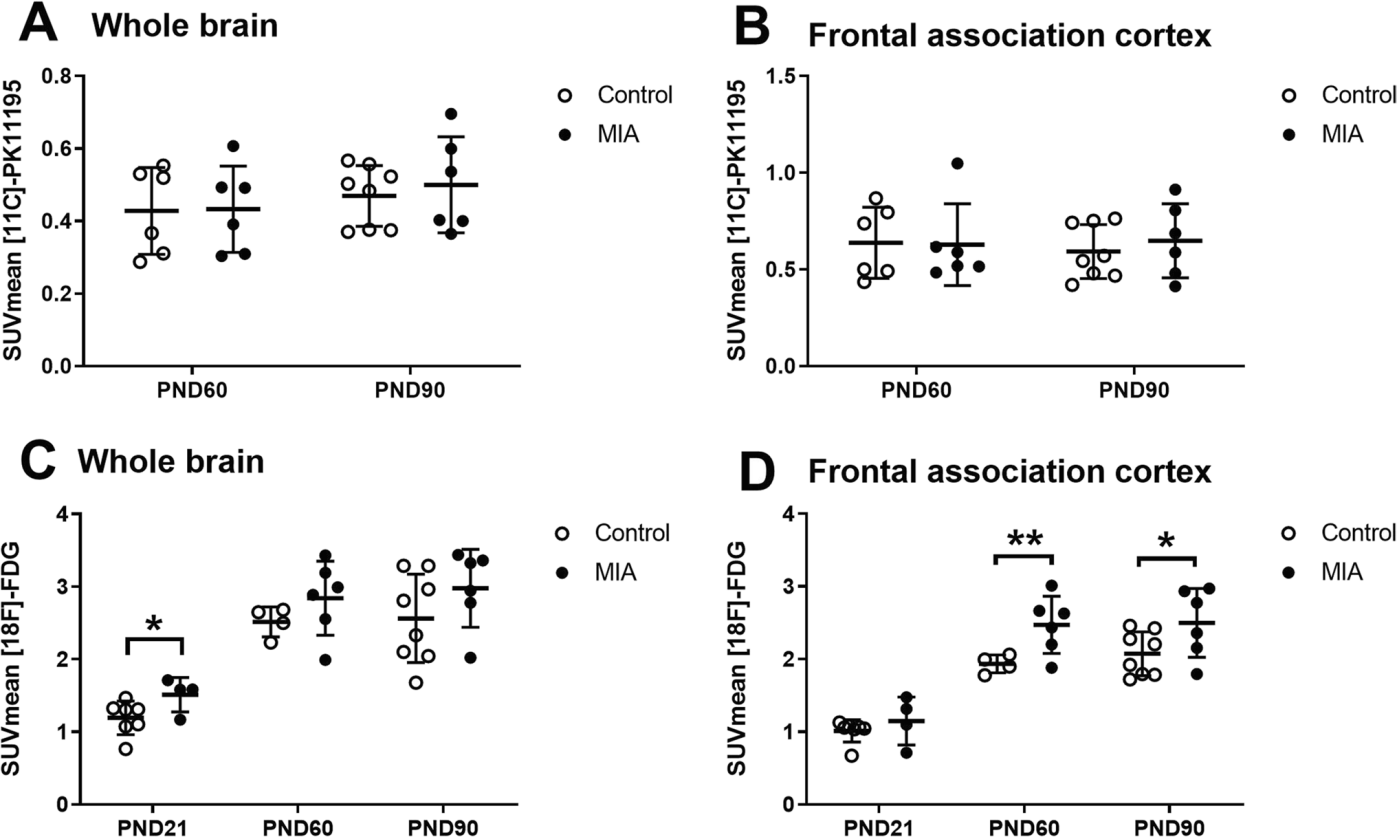
Effect of MIA on brain TSPO expression and cerebral glucose consumption**. A.** TSPO expression in the whole brain and the **B.** frontal association cortex (PND60: control: n = 6, MIA n = 8, PND90: control n = 6, MIA n = 6). **C.** Glucose consumption in the whole brain and the **D.** frontal association cortex (PND21: control: n = 7, MIA n = 4, PND60: control: n = 4, MIA n = 6, PND90: control n = 8, MIA n = 6). Values represent mean ± SD. Statistically significant differences between groups are indicated by asterisks: *P < 0.05, **P < 0.01. Significant differences between time points are not shown.

To determine the effect of MIA on microglial activation, [11C]-PK11195 PET was performed to measure TSPO expression on PND60 and PND90 (Fig. 3A and B). There was no significant difference between MIA and control offspring in tracer uptake in any brain region at PND60 or PND90 (supplementary table 1). Moreover, no significant within-group differences between time points were observed.

**Fig. 4.**
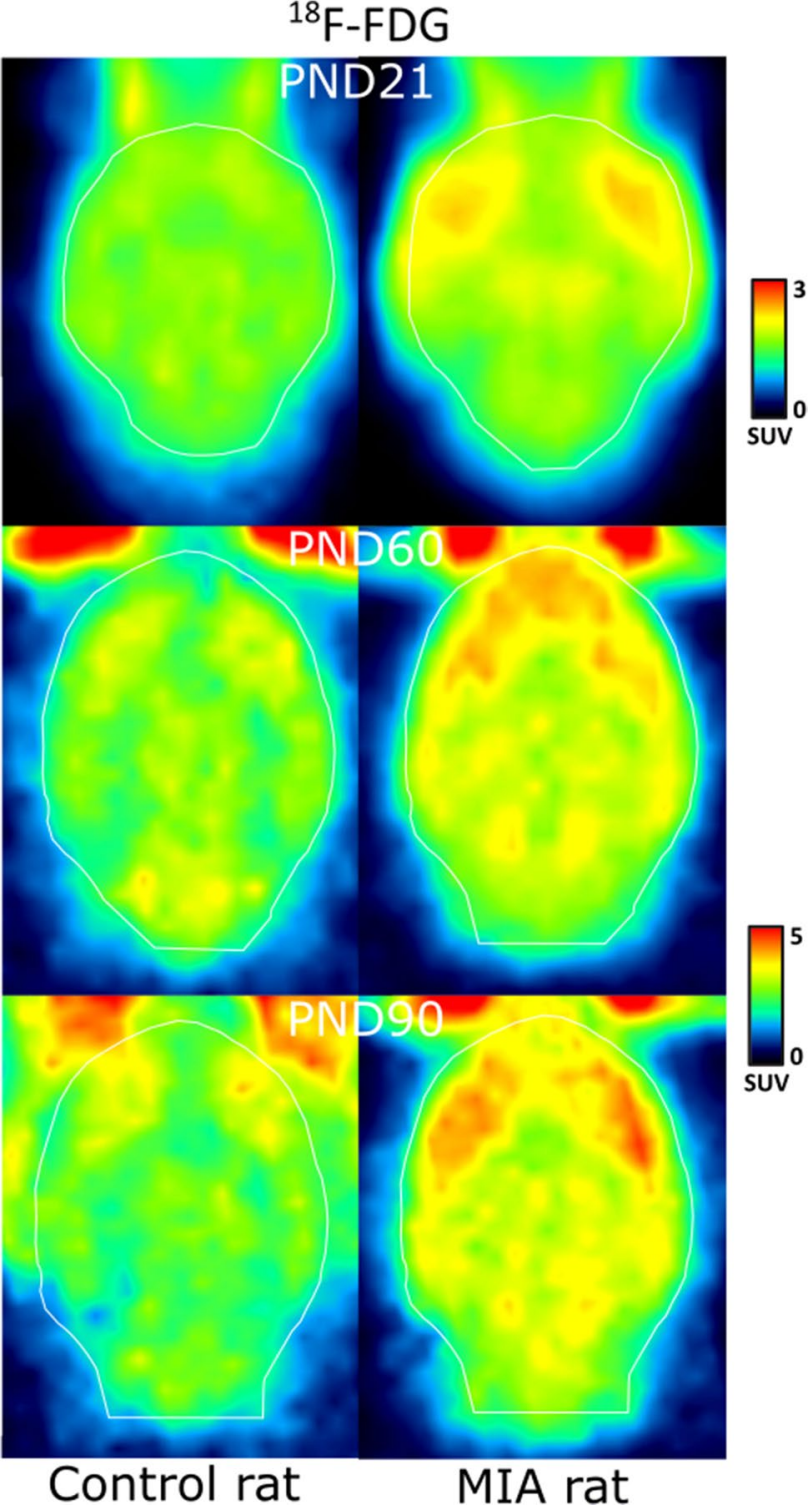
[18F]-FDG PET scan of a representative control and MIA rat on PND 21, 60, and 90.

### MIA Induced an Increase in Brain Glucose Consumption

To determine the effect of MIA on brain glucose metabolism, [18F]-FDG PET was performed on PND21, PND60 and PND90 (Figs. 3C, D and Fig. 4). A significant main effect of time was observed in all brain regions (p < 0.0001, Table 1), and a main effect of MIA in all brain regions (p < 0.05), except the entorhinal cortex (p = 0.052) and occipital cortex (p = 0.20). On PND21, MIA offspring had significantly higher tracer uptake in amygdala (p = 0.0001), BNST (p = 0.007), cerebellum (p = 0.0001), medial prefrontal cortex (p = 0.041), whole-brain (p = 0.016), frontal cortex (p = 0.048), thalamus (p = 0.037), brainstem (p = 0.007), basal ganglia (p = 0.027) and forebrain (p = 0.031) than control animals. On PND60, MIA offspring only had significantly higher tracer uptake in frontal association (p = 0.001) and frontal cortex (p = 0.045) than controls. On PND90, only the significant increase in tracer uptake in the frontal association cortex persisted (p = 0.037).

**Table 1.** [18F]-FDG PET: tracer uptake in the brain of control animals (control), and animals from mothers exposed to poly-I:C injection (MIA). Tracer uptake (SUV) is presented for different brain areas. Data are shown as mean ± SD. Statistically significant differences between MIA and control animals on the same day are indicated in bold and with an asterisk: *p < 0.05; **p < 0.01, ***p < 0.01

Brain regions	Main effect MIA (p-value)	PND21		PND60		PND90	
		Control	MIA	Control	MIA	Control	MIA
Amygdala	0.015	1.02 ± 0.16	1.31 ± 0.12***	2.19 ± 0.19	2.45 ± 0.49	2.17 ± 0.53	2.61 ± 0.44
BNST	0.001	1.31 ± 0.26	1.75 ± 0.31**	2.49 ± 0.25	2.98 ± 0.62	2.51 ± 0.62	3.08 ± 0.57
Cerebellum	0.026	1.01 ± 0.18	1.40 ± 0.13***	2.31 ± 0.15	2.55 ± 0.41	2.31 ± 0.54	2.59 ± 0.39
Corpus callosum	0.038	1.42 ± 0.32	1.77 ± 0.40	2.97 ± 0.28	3.33 ± 0.67	3.01 ± 0.77	3.45 ± 0.66
Entorhinal cortex	0.052	1.03 ± 0.17	1.22 ± 0.25	2.37 ± 0.21	2.68 ± 0.50	2.42 ± 0.59	2.81 ± 0.51
Frontal association cortex	0.001	1.01 ± 0.15	1.15 ± 0.33	1.9 ± 0.12	2.47 ± 0.39**	2.07 ± 0.30	2.50 ± 0.47*
Insular cortex	0.013	1.19 ± 0.20	1.50 ± 0.10	2.61 ± 0.25	3.01 ± 0.53	2.84 ± 0.63	3.41 ± 0.73
Medial prefrontal cortex	0.027	1.39 ± 0.30	1.82 ± 0.41*	3.03 ± 0.37	3.55 ± 0.71	3.19 ± 0.80	3.67 ± 0.78
Orbitofrontal	0.011	1.42 ± 0.29	1.76 ± 0.41	2.94 ± 0.29	3.55 ± 0.61	3.22 ± 0.72	3.74 ± 0.75
Occipital cortex	0.201	1.07 ± 0.22	1.24 ± 0.26	2.31 ± 0.18	2.47 ± 0.44	2.30 ± 0.57	2.53 ± 0.49
Nucleus accumbens	0.014	1.29 ± 0.23	1.64 ± 0.37	2.87 ± 0.29	3.28 ± 0.65	2.90 ± 0.76	3.46 ± 0.68
Striatum	0.011	1.43 ± 0.30	1.82 ± 0.42	2.87 ± 0.26	3.44 ± 0.70	3.04 ± 0.79	3.62 ± 0.71
Hippocampus	0.010	1.31 ± 0.29	1.59 ± 0.37	2.66 ± 0.25	3.07 ± 0.61	2.63 ± 0.64	3.17 ± 0.57
Whole brain	0.017	1.19 ± 0.23	1.51 ± 0.24*	2.51 ± 0.21	2.84 ± 0.51	2.56 ± 0.61	2.98 ± 0.54
Temporal cortex	0.028	1.12 ± 0.20	1.36 ± 0.27	2.45 ± 0.21	2.81 ± 0.49	2.60 ± 0.61	3.04 ± 0.58
Frontal cortex	0.010	1.12 ± 0.20	1.39 ± 0.27*	2.36 ± 0.20	2.7 ± 0.45*	2.44 ± 0.52	2.84 ± 0.54
Parietal cortex	0.044	1.14 ± 0.23	1.43 ± 0.30	2.46 ± 0.22	2.68 ± 0.44	2.53 ± 0.59	2.86 ± 0.54
thalamus	0.002	1.25 ± 0.26	1.63 ± 0.36*	2.50 ± 0.23	2.94 ± 0.59	2.57 ± 0.63	3.1 ± 0.55
midbrain	0.006	1.45 ± 0.33	1.81 ± 0.41	2.72 ± 0.26	3.07 ± 0.63	2.7 ± 0.64	3.2 ± 0.58
Brainstem	0.020	1.28 ± 0.28	1.64 ± 0.20*	2.54 ± 0.23	2.82 ± 0.53	2.52 ± 0.59	2.92 ± 0.46
Basal ganglia	0.003	1.25 ± 0.23	1.60 ± 0.31*	2.47 ± 0.20	2.93 ± 0.60	2.53 ± 0.65	3.07 ± 0.61
forebrain	0.002	1.26 ± 0.26	1.63 ± 0.35*	2.49 ± 0.22	2.93 ± 0.59	2.54 ± 0.63	3.08 ± 0.55

## Discussion

In this study, we found that prenatal immune activation increased brain glucose consumption and did not affect microglia activation. The increased glucose consumption in the frontal cortex of MIA offspring remained until adulthood and was associated with increased anxiety-like behaviour during adolescence and recognition memory deficits in adulthood.

Abnormal glucose metabolism is believed to be an indicator of underlying pathology. On PND21, MIA offspring displayed a higher glucose consumption, a specific aspect of metabolism, in the whole brain. Similarly, an [18F]-FDG PET study observed a shift in the increase in brain glucose consumption from PND18 to PND21 in the offspring of female rats exposed to the toxin methylmercury [34]. This suggests that MIA had a similar effect and delayed brain development. MIA may have induced a subtle change in the number and activity of microglia and increased activity of the complement system which could have resulted in altered neurodevelopmental processes, such as synaptogenesis, myelination, and synaptic pruning [35]. MIA was indeed shown to alter synaptic density in the offspring, which could be restored if an anti-inflammatory treatment was applied [36]. We, therefore, hypothesize that the increased brain glucose consumption observed on PND21 is a sign of altered neurodevelopment, which may have contributed to the development of behavioural alterations later in life.

MIA exposure is a validated experimental model that affects the negative, cognitive, and positive domains in rodents. In the negative domain, MIA offspring display anxiety-like behaviour as indicated by a reduced time spent in the open arms in the elevated-plus maze test or in the centre of the open field arena (OFT) [19, 21]. In our study, we found that MIA induced similar anxiety-like behaviour in the OFT. Regarding the cognitive domain and in accordance with previous studies [24, 25], we found that MIA induced recognition memory deficits during adulthood (PND90), as indicated by a reduced discrimination index in the NOR test. These results are consistent with clinical data indicating anxiety and impaired recognition memory in schizophrenia [37, 38] and other neurodevelopmental disorders such as autism [39, 40], and bipolar disorder [41, 42]. These behavioural changes were associated with increased brain glucose consumption in the frontal cortex. The frontal cortex is involved in the regulation of anxiety-like behaviour [43] and recognition memory as dysregulation of the connectivity between the frontal cortex and the hippocampus or basolateral amygdala results in anxiety [44, 45] and recognition memory deficits [46]. The frontal cortex is also crucial for other cognitive tasks, such as spatial discrimination memory, fear conditioning, and working memory; tasks shown to be impaired by prenatal immune activation [47–51]. Interestingly, in our study anxiety-like behaviour occurred before the cognitive deficits. It has been proposed that anxiety can further alter the frontal cortex, which in turn could deteriorate cognitive processes [52]. In the positive domain, prepulse inhibition, a measure of sensorimotor gating, is impaired in patients with neurodevelopmental disorders, such as schizophrenia [53], bipolar disorder [54], and autism [55]. Current literature is inconsistent as some studies observed PPI deficits in MIA offspring [18, 23, 56], while others did not [57, 58]. Ours results are in accordance with the latter as we did not observe change in PPI. The discrepancy between this study and others can be explained by the differences in dose and timing of the prenatal inflammatory challenge in different animal strains as these are known to affect the behavioural outcome [19, 59].

Other [18F]-FDG PET studies observed that MIA offspring displayed an increase in glucose consumption in the thalamus (PND35-100), globus pallidus (PND35-100), amygdala (PND100), and nucleus accumbens (PND100), and reduced brain glucose consumption in the ventral hippocampus and prefrontal cortex (PND35-100) [15, 16]. The most striking difference between our and other studies is the opposite direction of the change in glucose consumption in the frontal cortex. The discrepancy between our and other studies is unlikely to be due to a difference in the MIA protocol and species used as we intravenously injected a similar poly-I:C dose (4 mg/kg) in the same rat species and at the same gestational day (GD15). Possible explanations for the discrepancy are that the timepoint and analysis of the [18F]-FDG PET scans were different. While we measured absolute differences between groups using the SUV, other studies measured relative differences in tracer uptake using voxel-by-voxel comparisons after normalization to the average tracer uptake in the brain. Normalization of tracer uptake may have obscured group differences. Interestingly, similar discrepancies are also observed in clinical studies. Some clinical studies using [18F]-FDG PET, observed that unmedicated schizophrenia patients displaying positive symptoms had a hypermetabolic status in brain regions, such as the frontal cortex, thalamus, striatum, and temporal lobe [8, 60]. Such a hypermetabolic state could also be observed using [18F]-FDG PET in the psilocybin model of psychosis or following the injection of ketamine [61, 62]. On the other hand, other studies reported a decreased [18F]-FDG uptake in brain areas, such as the frontal cortex, in schizophrenia patients, which was associated with the negative symptoms of schizophrenia [7, 63, 64]. Thus, in schizophrenia patients, the positive and negative symptoms may be associated with increased and decreased brain glucose consumption, respectively.

Prenatal infection is believed to increase the risk of schizophrenia via priming of immune cells or causing neuroinflammation [65]. In our study, [11C]-PK11195 PET did not reveal changes in TSPO, a protein observed in reactive microglia and known to be involved in neuroinflammation on PND60 and PND90 in MIA offspring. Possibly MIA induced a subtle change in microglia activity, which could not be detected due to a lack of sensitivity of the [11C]-PK11195 PET tracer. Perhaps MIA primed, rather than activated the immune system, which would be in line with another study showing that exposure to an additional stressor during late adolescence in MIA offspring was necessary for inducing an inflammatory response [44]. Another possibility is that we did not scan at the correct timepoint. As the largest increase in [18F]-FDG uptake was on PND21, a similar increase could perhaps also have been observed using [11C]-PK11195 PET. However, this timepoint was not measured to avoid another challenging i.v. injection and anaesthesia in young animals and to reduce animal burden. The preclinical data on microglial changes in MIA offspring is contradictory. While some studies reported no change in microglial density and morphology [17, 58], others reported an increase in density of reactive microglia [66, 67] or even a decrease in reactivity in some brain regions [17, 66]. Many factors may explain such differences, including differences in the day of poly-I:C injection, injected dose, time of evaluation and strains used. A previous PET imaging study observed higher [11C]-PK11195 uptake in the prefrontal cortex and hippocampus in MIA offspring than in control rats [67]. They used the uptake ratio relative to the cerebellum as outcome parameter, and thus assumed no inflammatory changes in the cerebellum. A limitation of using the uptake ratio is that it does not show if an effect is caused by the target region or the reference region. Clinical imaging studies in schizophrenia patients observed either a light increase or no differences in microglial activation depending on the outcome parameter used [11].

Limitations of this study include a limited sample size in some of the [18F]-FDG imaging and behavioural tests, which may have prevented the observation of clear correlations between imaging and behavioural parameters in this dataset. However, despite not observing significant correlations, we observed a clear increase in brain glucose consumption on PND21, 60 and 90, anxiety-like behaviour on PND60, and recognition memory deficits on PND90. Another limitation is that we did not correct for multiple comparisons, therefore, the data should be interpreted with caution.

## Conclusion

Overall, this study suggests that prenatal immune activation changed early neurodevelopmental processes, eventually resulting in behavioural alterations later in life. These changes were accompanied by increased brain glucose consumption, but not reactive glial cells, from early life and throughout adolescence and adulthood. This increased brain glucose consumption was associated with alterations in anxiety-like behaviour and recognition memory during adolescence and adulthood, respectively.

## Supplementary Information

Below is the link to the electronic supplementary material.

Supplementary file1 (DOCX 182 KB)

Supplementary file2 (PDF 293 KB)
