# Supplementary material for: Immune Activation in Pregnant Rats Affects Brain Glucose Consumption, Anxiety-like Behaviour and Recognition Memory in their Male Offspring
Source: Mol Imaging Biol. 2022 Apr 5;24(5):740–9. doi: 10.1007/s11307-022-01723-3 (PMC9581871; doi:10.1007/s11307-022-01723-3)
Supplement: Supplementary file 1 — Supplementary file1 (DOCX 182 KB) [file 11307_2022_1723_MOESM1_ESM.docx]

Electronic Supplementary Material

**Immune activation in pregnant rats affects brain glucose consumption, anxiety-like behaviour and recognition memory in their male offspring**

Journal of Molecular Imaging and Biology

Cyprien G. J. Guerrin, Alexandre Shoji, Janine Doorduin, Erik F.J. de Vries

Department of Nuclear Medicine and Molecular Imaging, University Medical Center Groningen, University of Groningen, Hanzeplein 1, 9713, GZ, Groningen, the Netherlands.

Corresponding author: Prof. Dr. Erik F. J. de Vries, [e.f.j.de.vries@umcg.nl](mailto:e.f.j.de.vries@umcg.nl), [+31 50 361 3599](tel:+31503613599)

**Supplementary figure 1.** [^11^C]-PK11195 PET scan of a representative control and MIA rat on PND 21, 60, and 90.


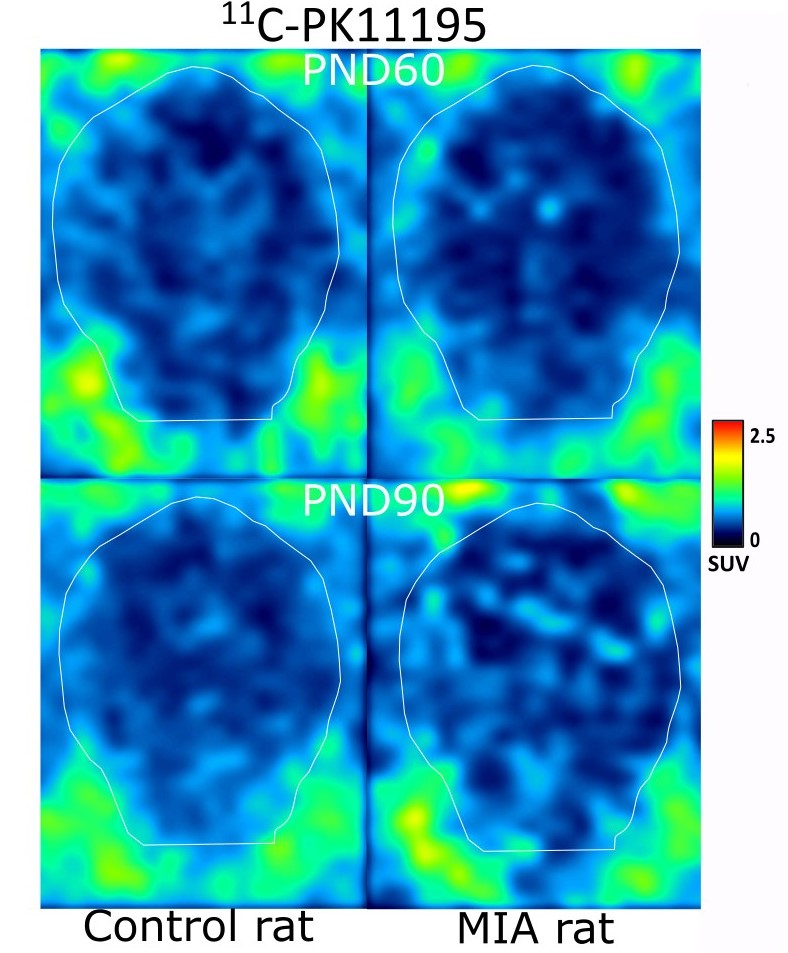


**Supplementary table 1. [^11^C]-PK11195 PET: tracer uptake in the brain** of control animals (control), and animals from mothers exposed to poly-I:C injection (MIA). Tracer uptake (SUV) is represented for different brain areas. Data are shown as mean ± SD. There was no statistically significant difference between MIA and control animals.

| **Brain regions** | **Main effect MIA**  **(p-value)** | **PND60** | | **PND90** | |
| --- | --- | --- | --- | --- | --- |
|  |  | **Control** | **MIA** | **Control** | **MIA** |
| Amygdala | 0.87 | 0.38±0.11 | 0.39±0.14 | 0.42±0.05 | 0.42±0.10 |
| BNST | 0.47 | 0.34±0.11 | 0.27±0.08 | 0.35±0.06 | 0.37±0.15 |
| Cerebellum | 0.23 | 0.49±0.14 | 0.55±0.15 | 0.55±0.14 | 0.64±0.15 |
| Corpus callosum | 0.45 | 0.34±0.09 | 0.36±0.10 | 0.40±0.08 | 0.44±0.14 |
| Entorhinal cortex | 0.27 | 0.44±0.13 | 0.48±0.14 | 0.46±0.07 | 0.52±0.11 |
| Frontal association cortex | 0.76 | 0.64±0.18 | 0.63±0.21 | 0.59±0.15 | 0.65±0.20 |
| Insular cortex | 0.79 | 0.46±0.13 | 0.47±0.14 | 0.47±0.08 | 0.48±0.11 |
| Medial PFC | 0.47 | 0.30±0.07 | 0.32±0.10 | 0.36±0.07 | 0.38±0.13 |
| Orbitofrontal | 0.48 | 0.40±0.11 | 0.41±0.13 | 0.40±0.09 | 0.45±0.13 |
| Occipital cortex | 0.96 | 0.57±0.16 | 0.55±0.13 | 0.65±0.15 | 0.66±0.24 |
| Nucleus accumbens | 0.63 | 0.31±0.08 | 0.32±0.10 | 0.36±0.08 | 0.39±0.12 |
| Striatum | 0.91 | 0.30±0.07 | 0.30±0.08 | 0.36±0.06 | 0.37±0.10 |
| Hippocampus | 0.51 | 0.32±0.10 | 0.34±0.11 | 0.38±0.07 | 0.41±0.11 |
| Whole brain | 0.70 | 0.43±0.12 | 0.43±0.12 | 0.47±0.09 | 0.50±0.14 |
| Temporal cortex | 0.41 | 0.45±0.12 | 0.48±0.14 | 0.46±0.08 | 0.51±0.11 |
| Frontal cortex | 0.79 | 0.52±0.14 | 0.48±0.13 | 0.48±0.12 | 0.53±0.18 |
| Parietal cortex | 0.95 | 0.51±0.15 | 0.48±0.11 | 0.53±0.11 | 0.55±0.19 |
| thalamus | 0.60 | 0.35±0.11 | 0.31±0.10 | 0.37±0.07 | 0.37±0.09 |
| midbrain | 0.72 | 0.33±0.10 | 0.34±0.12 | 0.40±0.08 | 0.42±0.11 |
| Brainstem | 0.49 | 0.39±0.12 | 0.42±0.14 | 0.45±0.08 | 0.48±0.12 |
| Basal ganglia | 0.62 | 0.30±0.09 | 0.30±0.10 | 0.34±0.05 | 0.37±0.11 |
| forebrain | 0.69 | 0.35±0.10 | 0.31±0.10 | 0.38±0.07 | 0.38±0.10 |
